# A Novel System for Identification of Inhibitors of Rift Valley Fever Virus Replication

**DOI:** 10.3390/v2030731

**Published:** 2010-03-18

**Authors:** Mary E. Piper, Sonja R. Gerrard

**Affiliations:** 1 Cellular and Molecular Biology Program, University of Michigan, Ann Arbor, MI, USA; E-Mail: mapipe@umich.edu (M.E.P.); 2 Department of Epidemiology, University of Michigan, Ann Arbor, MI, USA

**Keywords:** Rift Valley fever, virus-like particle, RNA virus, *Bunyaviridae*, Phlebovirus, minigenome, antivirals

## Abstract

Rift Valley fever virus (RVFV) is a human and livestock pathogen endemic to sub-Saharan Africa. We have developed a T7-dependent system for the efficient production of RVFV-like particles (RVF-VLPs) based on the virulent ZH-501 strain of RVFV. The RVF-VLPs are capable of performing a single round of infection, allowing for the study of viral replication, assembly, and infectivity. We demonstrate that these RVF-VLPs are antigenically indistinguishable from authentic RVFV and respond similarly to a wide array of known and previously unknown chemical inhibitors. This system should be useful for screening for small molecule inhibitors of RVFV replication.

## 1. Introduction

The Rift Valley fever virus (RVFV) is a vector and aerosol-borne virus endemic to sub-Saharan Africa. RVFV can cause disease in humans and in common livestock species, such as sheep and cattle. Pregnant animals exhibit a high incidence of abortion and young animals frequently succumb to infection [1]. People typically develop a flu-like illness, however, more severe manifestations, such as hemorrhagic fever or encephalitis, occur in a small percentage of cases. The hemorrhagic and encephalitic forms of disease exhibit high case-fatality rates [2–4]. Competent vectors of RVFV are found worldwide [5–12], and release of the virus into a new region would be damaging for both the economy and public health. In 2000, the first documented outbreak of the virus outside of the African continent occurred on the Arabian Peninsula, and the virus was determined to be of East African origin [13–14]. The presence of the virus outside of Africa demonstrates the ability of the virus to spread to previously unaffected regions of the world, and geographic isolation appears to be mainly responsible for the containment of the virus to African regions. No licensed vaccines or therapeutic treatments are available; our RVF-VLP system will aid in the screening of small molecule inhibitors for the development of novel therapeutics.

RVFV is an enveloped virus belonging to the *Phlebovirus*genus of the*Bunyaviridae*family. RVFV contains a negative-sense RNA genome that is divided into three segments, termed the small (S), medium (M), and large (L) segments. The S segment encodes the nucleocapsid protein (N), which encapsidates the viral RNA genome, and a nonstructural protein, NSs, which is involved in inhibition of the host innate-immune response [1]. The M segment encodes the envelope glycoproteins, Gn and Gc, and nonstructural proteins, termed NSm, while the L segment encodes an RNA-dependent RNA polymerase (RdRp). The nonstructural proteins, NSs and NSm are not required for viral replication in cell culture [15–18]. Transcription and replication of the viral genome is initiated through the recognition of promoter sequences in the 5’ and 3’ terminal untranslated regions (UTRs) of each genomic segment [19,20]. These processes occur in the cytoplasm and require both RdRp and N [1,21]. The RdRp and N are translated in the cytoplasm, while the envelope glycoproteins enter the secretory system. Gn and Gc form a complex and localize in steady-state to the Golgi apparatus due to a signal found on Gn [22–24]. The assembled virus buds into the lumen of the Golgi apparatus, and virions are released from the cell when elements of the Golgi fuse with the plasma membrane.

We have developed a T7 RNA polymerase (T7 RNAP)-dependent system for the production of infectious RVFV-like particles (RVF-VLPs) that obviates the need for high-level biosafety containment and allows for generation of viral particles that are antigenically indistinguishable from authentic RVFV. RVF-VLPs can deliver a minigenome to target cells and expression of RdRp and N in target cells is not necessary for generation of significant reporter activity. Minigenome activity is inhibited by the same chemical compounds that inhibit authentic RVFV replication. RVF-VLPs are efficiently produced in our system, which should allow for future studies aimed at examination of virus assembly and screening for small molecule inhibitors of viral entry and replication.

## 2. Results

### 2.1. System for Production of RVF-VLPs from ClonedcDNAs

**Figure 1:**
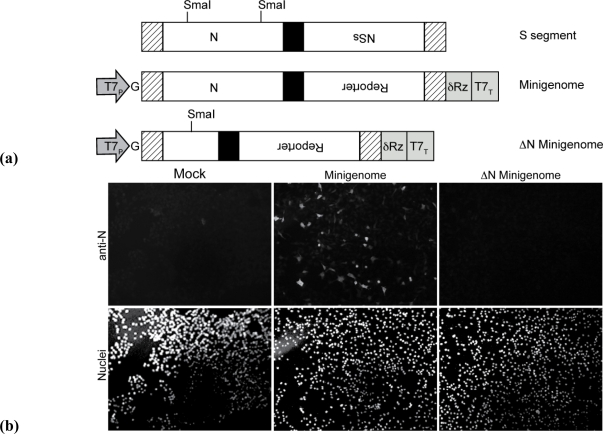

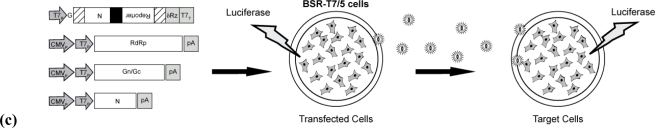
**(a)** *Schematic of RVFV S segment-based minigenome and procedure for generation and analysis of RVF-VLPs.* The minigenome plasmids are derived from the S-segment. The minigenome is flanked by a T7 promoter (T7P) and hepatitis delta ribozyme (δRz) and T7 terminator (T7T). T7 RNA polymerase (RNAP) initiates transcription at the final G residue in the promoter and terminates at the T7T site. Following transcription, the δRz excises itself to generate an authentic viral 3’ terminus. The 5’ and 3’ UTRs are indicated by hashed marks and the intergenic region is indicated in black. **(b)** *Internal deletion in N ORF prevents expression from* Δ*N minigenome.* BSR-T7/5 cells were either transfected with empty vector (EV) or transfected with minigenome or ΔN minigenome. Cells were fixed 24 h post-transfection and incubated with rabbit anti-N polyclonal antibody, followed by Alexa Fluor 488 mouse anti-rabbit secondary antibody. Slides were mounted in Prolong antifade with Dapi. **(c)** *Schematic of RVF-VLP production.* Minigenome along with expression plasmids for N, RdRp, and Gn/Gc are transfected into BSR-T7/5 cells. The expression constructs have the open reading frames downstream of T7 (T7_P_) and CMV promoters (CMV_P_) and are followed by polyadenylation signals (pA), generating high-level constitutive expression of the genes. Theminigenome is first transcribed by T7 RNAP followed by replication and transcription of the RNA by the RdRp and N. Transcription of the reporter gene on the minigenome results in production of the reporter molecule (RLuc or GFP). Expression of Gn and Gc results in packaging of the minigenome into RVF-VLPs that can be harvested and used to infect target cells. In target cells the minigenome is transcribed by the packaged RdRp, resulting in expression of the reporter molecule.

We utilized a set of plasmids similar to that described for rescue of the virulent ZH-501 strain of RVFV [16] as the basis of our RVF-VLP system (Table 1). In order to assess production of RVF-VLPs, a minigenome based on the S segment was generated such that the NSs open reading frame was replaced with a gene encoding a reporter molecule, such as GFP orrenillaluciferase (RLuc) (Figure 1a). Additionally, the vector backbone of all transcription plasmids was changed to one with transcriptional silencers flanking the cloning site (see Methods section). The new vector dramatically reduced the background expression of the reporter gene (data not shown). An additional transcription plasmid was constructed that lacked 237 nucleotides of the N gene (Figure 1a). Immunofluorescence microscopy (Figure 1b) and immuno-blot (data not shown) using a polyclonal antibody recognizing full-length N, failed to detect N expression. These S segment-based plasmids will henceforth be referred to as minigenome plasmids.

A schematic detailing our method for RVF-VLP production and analysis is shown in Figure 1c. The minigenome along with expression plasmids for N, RdRp, and Gn/Gc are transfected into BSR-T7/5 cells (Figure 1c). The BSR-T7/5 cells constitutively express T7 RNAP, which drives production of the primary minigenome transcript. Expression of the reporter from the minigenome in transfected cells requires co-expression of RdRp and N. Co-expression of Gn/Gc results in the production of RVF-VLPs containing the minigenome (Figure 1c). The RVF-VLPs are released from the cells into the media, and are then used to infect target cells (Figure 1c). Replication of the minigenome and expression of RLuc or GFP in RVF-VLP-infected target cells relies on packaging of the encapsidatedminigenome and RdRp. The RVF-VLPs do not contain the L or M genomic segments. Additionally, the S segment-based minigenome lacks the NSs gene and, in some experiments, the N gene. Therefore, RdRp, Gn/Gc, and in some cases, N, cannot be synthesized in infected target cells and thus further production of RVF-VLPs is prevented.

### 2.2. Production of Infectious RVF-VLPs

We investigated the ability of the recombinant structural proteins, N, RdRp and Gn/Gc, to replicate and transcribe the minigenome in transfected BSR-T7/5 cells. Replication of the minigenome requires expression of N and RdRp, but not Gn/Gc[21]. Cells transfected with minigenome and pN, but not pRdRp and pGn/Gc were unable to transcribe the minigenome reporter (Table 2, EV/EV), and the RLuc activity in these samples was considered background. The addition of pRdRp resulted in RLuc activity at levels greater than 1,500-fold background levels (Table 2, RdRp/EV). Though Gn/Gc is not required for replication of the minigenome, Gn/Gc expression further increased the production of RLuc in transfected cells to levels greater than 40,000-fold background (Table 2, RdRp/Gn/Gc). The increase in RLuc expression could be due to an effect of Gn/Gc on RdRp activity or due to RVF-VLP-infection of other cells in the monolayer.

RVF-VLPs released into the media from the cells transfected in Table 2 were used to infect various target cells, and RLuc expression in the RVF-VLP-infected target cells was measured. Cells receiving media from cells transfected with minigenome, pN, pRdRp (RdRp/EV) did not generate RLuc expression greater than background for any timepoint or cell type investigated. By contrast, target cells receiving media from cells transfected with minigenome, pN, pRdRp, and pGn/Gc (RdRp,Gn/Gc) produced RLuc activity substantially above background for all timepoints and cell types (Table 3). Therefore, infectious RVF-VLP production is dependent on expression of Gn/Gc. The production of RVF-VLPs peaked at 48 h post-transfection, however considerable amounts of RVF-VLPs were released at 72 h post-transfection (Table 3).

RLuc activity in RVF-VLP-infected target cells did not require expression in *trans* of the T7 RNAP or RdRp and N. RVF-VLP-infection of Vero E6 cells, which do not express the T7 RNAP or any viral proteins, produced RLuc levels that were over 200-fold background (Table 3). However, the addition of support plasmids did increase RLuc activity in BSR-T7/5 and Vero E6 cells. For instance, at the 48 h timepoint, expression of RdRp and N in BSR-T7/5 cells increased RLuc activity greater than 15-fold and expression of RdRp in Vero E6 cells increased RLuc activity 1.8-fold (Table 3).

### 2.3. RVF-VLPs are Efficiently Produced

**Figure 2:**
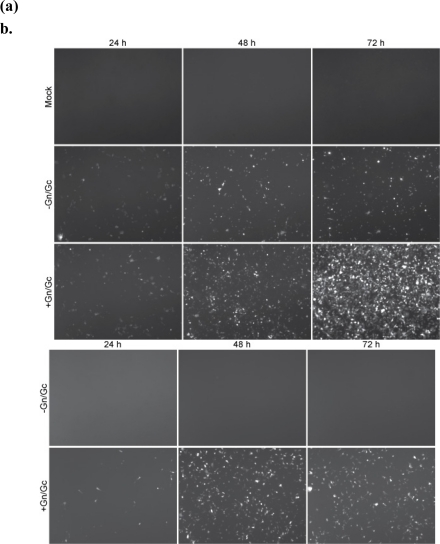
**(a)** Cells were transfected with the GFP minigenome, pN and either empty vector (EV/EV), pRdRp and empty vector (RdRp/EV), or pRdRp and pGn/Gc (RdRp/Gn/Gc) and analyzed at the indicated times for expression of GFP. **(b)** Media from cell monolayers shown in (a) was harvested at the indicated times and used to infect BSR-T7/5 cells that expressed RdRp and N.

Using the green fluorescent protein (GFP) version of the minigenome, we investigated whether the increase in RLuc activity in transfected cells due to expression of Gn/Gc (Table 2) was caused by RVF-VLP infection of cells in the transfected cell monolayer. BSR-T7/5 cells transfected with the GFP minigenome, pN, and either empty vector (EV/EV), pRdRp and empty vector (RdRp/EV), or pRdRp and pGn/Gc(RdRp/Gn/Gc), were visualized by fluorescence microscopy (Figure 2a). As expected, no GFP signal was detected in cells that lacked RdRp and Gn/Gc (Figure 2a, EV/EV). However, in cells that expressed RdRp (RdRp/EV), or RdRp and Gn/Gc (RdRp/Gn/Gc) GFP expression was evident (Figure 2a). Although the signal intensity increased over time in cells that lacked Gn/Gc, the percentage of cells expressing GFP did not increase (Figure 2a). With addition of the glycoproteins (RdRp/Gn/Gc), the intensity of GFP fluorescence as well as the percentage of cells expressing GFP increased over time (Figure 2a). Therefore, it appears that the increase in RLuc activity observed in the experiment shown in Table 2 is mainly due to spread of RVF-VLPs in the transfected cell monolayer

The media harvested from the transfected cells (Figure 2a) at 24, 48, or 72 h post-transfection was placed onto target cells and GFP expression was visualized by fluorescent microscopy (Figure 2b). The passage from cells lacking Gn/Gc did not produce any GFP, while passage from cells expressing Gn/Gc did exhibit GFP expression in target cells. The number of cells expressing GFP and the intensity of GFP expression was greatest for cells infected with RVF-VLPs (RdRp/Gn/Gc) harvested 48 h post-transfection. However, RVF-VLP production appeared to exhibit high yields at 72 h post-transfection, mimicking the RLuc results shown in Table 3. The majority of the cells in the monolayer appeared to express GFP after RVF-VLP (RdRp/Gn/Gc) infection, demonstrating that RVF-VLPs were efficiently produced.

### 2.4. RVF-VLPs are Antigenically Indistinguishable from Authentic RVFV

**Figure 3:**
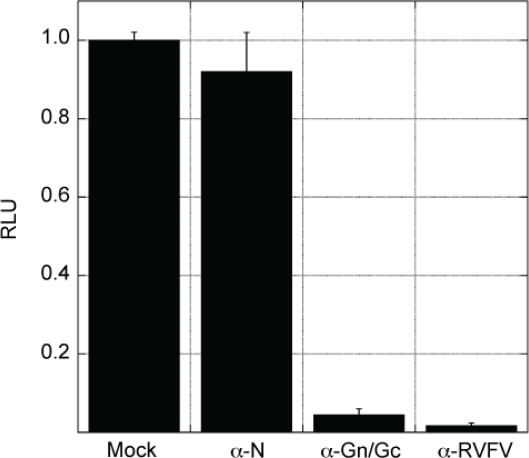
*RVF-VLPs are neutralized by the same antibodies that neutralize RVFV.* RVF-VLPs that were generated and subjected to a 30 min incubation at room temperature with no antibody, monoclonal anti-N, neutralizing anti-Gn and anti-Gcmonoclonals or a polyclonal anti-RVFV antibody prior to application on target cells. The antibody concentration effective for neutralization was determined on authentic RVFV. The target cells were harvested at 24 h post-infection and the RLuc activity levels were measured. RLuc values (RLU) are expressed relative to the no antibody control. Shown is the data for a representative experiment performed in triplicate with standard deviation bars.

BSR-T7/5 cells were transfected with minigenome, pN, pRdRp, and pGn/Gc. The media containing RVF-VLPs was harvested and clarified, then, incubated with antibodies recognizing RVFV or not incubated with antibody (Mock). The media was then transferred to BSR-T7/5 target cells and RLuc activity was measured at 24 h post-infection. The level of RLuc activity in cells infected by RVF-VLPs receiving the Mock treatment represents 100% infectivity (Figure 3). Incubation of the RVF-VLPs with neutralizing polyclonal antibodies to RVFV nearly completely neutralized the RVF-VLPs, and resulted in minigenome activity levels that were only 1% of the Mock treatment (Figure 3). Incubation with neutralizing monoclonal antibodies recognizing the envelope glycoproteins [25], which are exposed on the surface of the RVF-VLPs, neutralized the RVF-VLPs dramatically, allowing only 6% activity (Figure 3). By contrast, monoclonal antibodies recognizing N, which is not exposed on the surface of the RVF-VLPs, did not significantly reduce infectivity of the RVF-VLPs (Figure 3). These antibodies were evaluated on the same strain of RVFV (ZH-501) that was used to generate our VLP system (data not shown), and the same trends were observed. Our results suggest that the RVF-VLPs are antigenically similar to virulent RVFV.

### 2.5. RVF-VLPs are Efficiently Harvested by High-Speed Centrifugation

**Figure 4:**
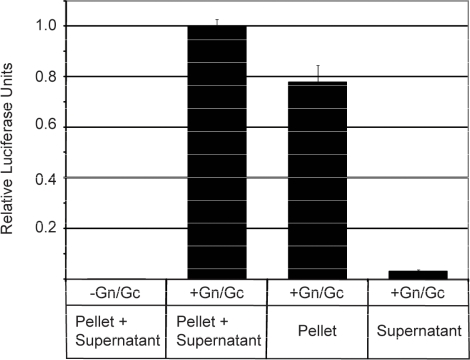
*RVF-VLPs can be harvested using high-speed centrifugation*. Clarified media from transfected cells was subjected to high-speed centrifugation as described in the materials and methods. The “Supernatant”,“Pellet” or “Pellet + Supernatant” were used to infect target cells. RLuc activity was measured at 24 h post-infection and is expressed in relative RLuc units. Shown is the data for a representative experiment performed in quadruplicate, the error bars reflect the standard deviation.

We devised a method for harvesting the RVF-VLPs in order to assay cellular release of particles and to determine the protein content of RVF-VLPs. BSR-T7/5 cells were transfected with minigenome, pN, pRdRp, and either pGn/Gc or empty vector. At 48 h post-transfection, the media from transfected cells was harvested, clarified, and RVF-VLPs were pelleted by ultracentrifugation. The concentrated RVF-VLPs in the pellet were either resuspended in the supernatant (Figure 4; Pellet + Supernatant) or the supernatant was decanted (Figure 4; Supernatant) and the pellet was resuspended in equivalent amount of fresh media (Figure 4; Pellet). These samples were used to infect BSR-T7/5 cells that expressed RdRp and N. At 24 h post-infection, the cells were harvested and RLuc activity was measured. RLuc activity in BSR-T7/5 cells that were infected with the passage containing both the supernatant and pellet fractions represents 100% infectivity. Nearly 80% of the infectivity was present in the pellet, while only 3% of the infectivity was in the supernatant. Presumably, the decrease in RLuc activity between the “Pellet and Supernatant” and “Pellet” samples is due to RVF-VLP loss when the media was decanted.

### 2.6. RVF-VLPsBehave Similar to Authentic RVFV with Respect to Small Molecule Inhibitors

We tested a panel of small molecule inhibitors for activity against RVF-VLPs and RVFV. Two of these compounds, ribavirin[26–29] and actinomycin D[30], had previously been tested for activity against RVFV and thus serve as positive controls. Ribavirin is a broad spectrum antiviral that is believed to inhibit viral replication either by directly acting on the RdRp or indirectly through inhibition a cellular enzyme necessary for biosynthesis of guanine nucleotides [31]. Actinomycin D was investigated due to its ability to inhibit cellular DNA-dependent RNA polymerases but not viral RNA-dependent RNA polymerases [32]. Guanidine and mycophenolic acid were tested because they have been shown to have activity against other RNA viruses. Guanidine is active against the RdRp of poliovirus and many other positive-sense RNA viruses [33] and has been also been shown to inhibit the RdRp of a dsRNA virus [34]. Mycophenolic acid acts on the same cellular enzyme as ribavirin [31,35]. And finally, monensin and ammonium chloride block the acidification of endocytic organelles [36]. The acidic organelles are required for membrane fusion for many viruses that enter host cells through receptor-mediated endocytosis.

**Figure 5:**
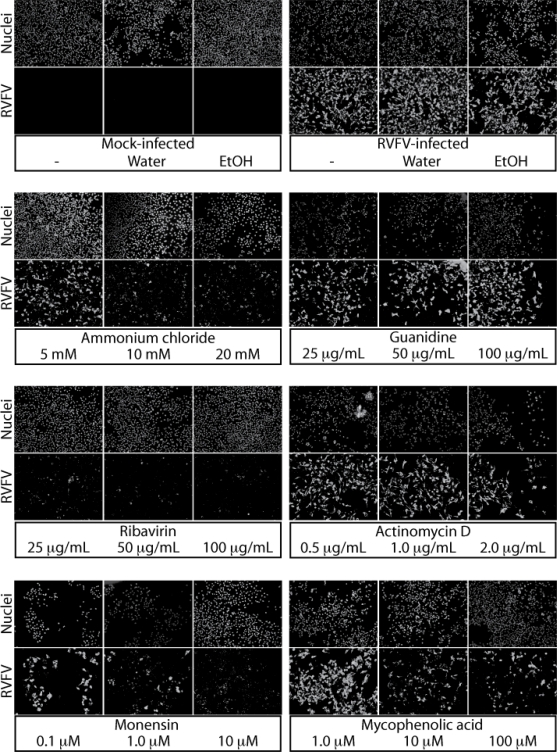
*Effect of small molecule inhibitors on RVFV replication*. Inhibitors were added at the indicated concentrations at the time of infection with the ZH548-MP12 vaccine strain of RVFV at an MOI of 1. At 22 h post-infection, cells were fixed and stained with rabbit anti-N followed by anti-rabbit Alexa 488. The nuclei were visualized with Dapi.

The inhibitors were added to the cultures at the time of infection with either RVF-VLPs or RVFV. Infected cells were harvested at 22 h post-infection and minigenome activity or expression of N was assayed, respectively. As expected, ribavirin was found to be a potent inhibitor of RVF-VLPs (Table 4) and RVFV (Figure 5). Mycophenolic acid was also a strong inhibitor (Table 4, Figure 5), suggesting that inhibition of guanine nucleotide biosynthesis is sufficient for inhibition of viral replication. The endocytic inhibitors, ammonium chloride and monensin, inhibited both RVF-VLPs (Table 4) and RVFV (Figure 5), suggesting that the virus enters cells through receptor-mediated endocytosis. Guanidine did not strongly inhibit either RVF-VLPs (Table 4) or RVFV (Figure 5). Actinomycin D blocked activity of RVF-VLPs (Table 4) but did not inhibit RVFV (Figure 5). Recently, Ikegami *et al.* discovered the combination of RVFV (MP12 strain) replication and host transcriptional repression, either by actinomycin D or RVFV NSs, was sufficient to induce the activation of protein kinase R (PKR) [37]. PKR inhibits translation of host and viral proteins, but can be targeted for degradation by RVFV NSs [30,37]. The RVF-VLPs do not express NSs, so the induction of PKR by actinomycin D cannot be down-regulated.

## 3. Discussion

In this paper we report on the development of a T7-dependent system for production of RVF-VLPs and its application to high-throughput screening for antivirals against RVFV. We are able to generate RVF-VLPs through the expression of a minigenome and four viral structural proteins;Gn, Gc, N and RdRp. Although these particles lack the full complement of genomic segments, they behave in a similar fashion to authentic RVFV in the four respects tested in this study. (1) RVF-VLPs are able to package a minigenome based on the S segment and deliver it to naive target cells. (2) RVF-VLPs are secreted from the cell and can be concentrated from the media by centrifugation. (3) RVF-VLPs are inhibited from infection of a naive monolayer by antibody neutralization using the same set of antibodies that neutralize RVFV. (4) Chemical inhibitors of RVFV replication also inhibit RVF-VLPs indicating that the minigenome activity is an appropriate surrogate for viral replication.

Plasmid-based genetic and RVF-VLP systems are powerful tools for studying the replicative cycle of viruses. We have modified the design of the T7 RNAP-driven plasmid-based rescue system for the virulent ZH-501 RVFV strain [16] in order to produce RVF-VLPs capable of performing only a single round of infection. In our design, expression plasmids encoding the open-reading frames of the structural proteins were expressed along with a minigenome. The expression plasmids do not contain the 5’ and 3’ UTRs that have been demonstrated to be required for packaging of the genome [19,20]. Thus, the RNAs produced from these plasmids cannot be packaged and these RVF-VLPs are capable of only a single round of replication. Using our system, we efficiently generated infectious RVF-VLPs.

Detection of minigenome activity in target cells was robust, and did not require expression of any RVFV proteins or T7 RNAP. Therefore, RVF-VLPs can be used to deliver minigenome to cells that are not efficientlytransfected, such as mosquito cells. Minigenome activity in RVF-VLP-infected target cells was reduced to near background levels by ribavirin, mycophenolic acid, ammonium chloride and monesin, all of which were also shown to be active against RVFV. Mycophenolic acid, ammonium chloride and monensin had not been tested previously for activity against RVFV. The results obtained with ammonium chloride and monensin, suggest that RVFV enters cells through receptor-mediated endocytosis. Our results are the first to examine endocytic entry by RVFV in the context of authentic RVFV infection. However, previous studies exploring the ability of the RVF envelope glycoproteins to form syncitia using either baculovirus or alphavirus-driven expression systems have previously found syncitia formation to be pH dependent [38,39]. Interestingly, we saw a differential effect with actinomycin D, in that this compound inhibits RVF-VLPs but not RVFV. Recently it has been shown that NSs from either MP12 (vaccine) or ZH-548 (virulent) strains is capable of degrading PKR [30,37]. Thus, it is likely that the differential effect we see with actinomycin D is because RVF-VLPs do not express NSs. However we cannot rule out the possibility that the differential effect of actinomycin D is the result of an inherent difference between how infected cells respond to RVFV versus RVF-VLPs.

Taken together our results strongly suggest that RVF-VLPs may serve as effective screening tools for identification of antivirals with activity against RVFV. The RVF-VLPs obviate the need for operating under select agent guidelines and BSL-3Ag conditions, which would be necessary if using virulent RVFV. Our replicon assay produced activity levels over 1,500-fold background, while our signal-to-noise values for the minigenome delivered by RVF-VLPs were found to be as high as 7,800. This value was obtained without any concentration of RVF-VLPs. Therefore, we have highly sensitive assays for both replication/transcription and RVF-VLP infectivity. Additionally, the RVF-VLPs express RLuc, thus they provide for a method of determining inhibition in a format that can be scaled to high-throughput levels. Furthermore, while the vaccine strain of RVFV (MP12) is cytopathic and can be used for high-throughput screening under BSL2 conditions, RVF-VLPs have the advantage of allowing for screening forsmall molecules that inhibit discreet viral processes and the ability to identify molecules that increase replication. For instance, RVF-VLPs can be used to screen specifically for effects on replication or entry into cells, thus making it easier to identify the target of inhibitory or enhancing molecules identified in a screen. Additionally, our RVF-VLPs are based on a virulent strain of RVFV, thus eliminating the potential of attenuating mutations influencing the activity of compounds.

We are using the RVF-VLPs system to study various steps in the RVFV replicative cycle, including entry, replication, assembly, and budding. The RVF-VLP system can be used to identify the viral proteins and genome elements necessary for the production of infectious RVF-VLPs, as well as elucidate the role of individual protein domains. Since expression of N or RdRp in target cells is not required for detection of RVF-VLP infection, we can analyze the effect of mutation on all of the structural proteins in order to identify protein-protein and protein-RNA interactions that are essential to virus assembly. Identification of critical protein domains will allow us to not only screen, but also design small molecule inhibitors targeting these important regions for the development of specific therapeutics.

## 4. Experimental Section

### 4.1. Plasmids

The construction of pTrRVFV-SΔNSs::GFP, pSTrRVFV-ΔNSmM, pN-Amp, and pGn/Gc have been described elsewhere[16,22]. pSTrRVFV-SΔNSs::hRLuc was derived from pTrRVFVSΔNSs::GFP in several steps. First, the GFP gene was released by digestion with EcoRV, followed by ligation with a humanized renillaluciferase gene (RLuc) that was flanked by EcoRV sites. The resulting plasmid was then subcloned into pSMART HC Kan (Lucigen). pSTrRVFVSΔNΔNSs::hRLuc was derived from pSTrRVFV-SΔNSs::hRLuc by removing the 237 nucleotide SmaI fragment. pRdRp-Amp (pSRG309) is derived from the L segment plasmid, pTrRVFV-L [16]. In brief, the RdRp ORF was amplified from pTrRVFV-L with primers that containedSalI (5’) and NotI (3’) sites. The resulting PCR product was then cloned into the SalI/NotI site of pIRES (Clontech) and the IRES was subsequently removed by digest of the plasmid with XhoI and SalI. The expression plasmids pN and pRdRp were constructed by cloning the open reading frames for N and RdRpinto pVAX1 (Invitrogen) using HindIII/EcoRI and BamHI/NotI sites, respectively.

### 4.2. Cells and Virus

Vero E6 and BSR-T7/5 cells were generous gifts from Dr. C. Fulhorst (University of Texas Medical Branch, Galveston) and Dr. K. Conzelmann (Max-von Pettenkofer-Institut, Munchen, Germany), respectively. BSR-T7/5 cells were subsequently cloned by limiting dilution and the resulting clonal lines were tested using the RVFV minigenome that expresses RLuc. Lines that produced high levels of RLuc from the minigenome were expanded. The C3 clone of the BSR T7/5 line was used for all experiments. The BSR-T7/5 and Vero E6 cells were grown in Dulbecco’s Modified Eagle Medium (Invitrogen) supplemented with 10% FCS and sodium pyruvate. The T7 RNAP transgene in the BSR-T7/5 cells was selected for using 1 mg/mLGeneticin (Invitrogen). The Vero E6 cells that stably express the RVFV RdRp (Vero E6-RdRp) were generated by transfection of Vero E6 cells with pSRG309 and pcDNA-Hygromycin (Invitrogen), then selection of hygromycin resistant cells with 200μg/mL hygromycin (Invitrogen). The ZH548-MP12 vaccine strain of RVFV used for all experiments involving infectious virus and was obtained from Dr. R. Tesh (World Reference Center of Emerging Viruses and Arboviruses).

### 4.3. Antibodies

Hybridomas that secrete neutralizing monoclonal antibodies that recognize Gn and Gc (R1-4B6-1-2, R1-4D4-1-1 and R5-3G2-1A) and monoclonal antibodies recognizing N (R1-P6-F6-6-2-2, R1-P6-F6-10-1-1, R1-P5-A6-12-2-2, RV-V-1B9-1-1, R3-1D8-1-2 and RV5-V6E4-1-1) were a generous gift of Dr. G. Ludwig (USAMRIID). Polyclonal antibodies that were generated against RVFV in mice were a generous gift of Dr. P. Rollin (CDC). Full-length N was expressed with an N-terminal histidine tag and purified under denaturing conditions on a Ni-NTA agarose column (Qiagen Inc.). The N antibody was generated in rabbits using purified protein as antigen (Harlan Laboratories). The secondary antibody used in immunofluorescence experiments was Alexa Fluor 488 goat anti-rabbit (Molecular Probes).

### 4.4. Virus-Like Particle Production

BSR-T7/5 cells were plated at 1 x 10^5^ cells/well in 12-well culture plates. After 24 h, cells were transfected using 2 μL/μgTransIT LT1 (Mirus Corporation) and plasmids in the ratio 0.25 μg minigenome: 0.50μg pN: 0.75 μg pRdRp: 0.50 μg pGn/Gc. Media on transfected cells was replaced every 24 h. Media containing RVF-VLPs was typically harvested at 48 or 72 h post-transfection, clarified by low speed centrifugation (300 rcf for 10 min at 4°C) and then diluted prior to being used to infect target cells. For some experiments target cells were transfected with pRdRp and pN 24 h prior to infection. Target cells were harvested at 24 h post-infection, and were analyzed by either fluorescence microscopy or RLuc assay (Promega). For some experiments, RVF-VLPs were concentrated. In those cases, clarified media was centrifuged at 82,700 rcf for 4 h at 4°C. The supernatant was removed and the pellet was resuspended in complete media.

### 4.5. RVFV Inhibitor Screen

Concentrated stocks of inhibitors were prepared either in water and filter sterilized (ribavirin, ammonium chloride, guanidine) or 100% ethanol (monensin, mycophenolic acid, actinomycin D). The inhibitors were obtained from Sigma-Aldrich (monensin, actinomycin D, guanidine), Calbiochem (mycophenolic acid), VWR (ammonium chloride) and RPI Corporation (ribavirin). RVF-VLPs and the ZH548-MP12 vaccine strain of RVFV (at an MOI of 1) were diluted 1:1 with 2X concentration of inhibitors in complete media. After 22 hours incubation, the inhibitors were removed and cells were analyzed for RLuc expression (RVF-VLPs) or immune fluorescence microscopy (RVFV-infected).

### 4.6. Immunofluorescence Microscopy

Cells were plated on glass coverslips and were fixed using freshly prepared 4% paraformaldehyde (Sigma-Aldrich) for 30 minutes. The paraformaldehyde was removed and cells were washed with PBS containing 1% BSA (PBS/BSA). The cells were permeabilized with PBS/BSA containing 0.1% Triton-X100 (Shelton Scientific, Inc.) for 30 minutes, then washed with PBS/BSA before adding the primary antibody. The primary antibody in all experiments was rabbit anti-N. The cells were washed again, and the secondary antibody goat anti-rabbit A488 (Molecular Probes) was added and incubated in the dark for 1 h. Finally, the cells were washed thoroughly and mounted onto glass slides using Prolong Antifade Gold with Dapi (Molecular Probes). The fluorescence was visualized using an Olympus BX-51 microscope in the University of Michigan Microscopy and Image Analysis Laboratory.

## 5. Conclusions

Our T7 RNAP-dependent VLP system efficiently generates RVF-VLPs, which are antigenically indistinguishable from authentic virus and behave similarly to chemical inhibitors. The sensitivity and design of the RVF-VLP system should allow for analysis of small molecule inhibitors of RVFV replication and entry in high-throughput format.

## Figures and Tables

**Table 1:** Plasmids used in this study.

**Plasmid name**	**Description**	**Ref**
pTrRVFV-SΔNSs::GFP	S segment-based minigenomeplasmid. The NSs gene has been replaced with GFP. Primary transcription is mediated by T7 RNAP. Vector backbone derived from pSP64 (Promega).	[16]
pRdRp	RdRp ORF cloned into pVAX1 (Invitrogen). Contains T7 RNAP and CMV promoters.	This study
pRdRp-Amp (pSRG309)	RdRp ORF cloned into pIRES (Clontech). Contains T7 RNAP and CMV promoters.	This study
pN	N ORF cloned into pVAX1 (Invitrogen). Contains T7 RNAP and CMV promoters.	This study
pSTrRVFV-SΔNSs::hRLuc	S segment-based minigenomeplasmid. The NSs gene has been replaced with hRLuc. Primary transcription is mediated by T7 RNAP. Vector backbone is pSMART HC Kan (Lucigen).	This study
pSTrRVFVSΔNΔNSs::hRLuc	Derived from pSTrRVFV-SΔNSs::hRLuc, the SmaI fragment within the N gene has been removed.	This study
pGn/Gc	Gn/Gcpolyprotein ORF cloned into pcDNA1.1	[22]

**Table 2:** Gn/Gc increases RLuc expression in transfected cells.

**Sample**	**Average** **Log Raw Luciferase Units (RLU)**
EV/EV	3.76
RdRp/EV	6.94
RdRp/Gn/Gc	8.37

**Table 3:** RdRp expression in *trans* enhances RLuc expression in target cells.

**VLP Harvest (hpt)**	**Sample**	**BSR-T7/5 (Log RLU/mL)**	**BSR-T7/5 RdRp/N-expressing (Log RLU/mL)**	**Vero (Log RLU/mL)**	**Vero RdRp-expressing (Log RLU/mL)**
24	EV/EV	3.21	3.71	3.22	3.24
	RdRp/EV	3.25	3.55	3.22	3.23
	RdRp/Gn/Gc	5.38	6.36	4.50	4.93
48	RdRp/EV	3.80	4.42	3.69	3.70
	RdRp/Gn/Gc	7.08	8.31	6.15	6.41
72	RdRp/EV	3.28	4.01	3.23	3.26
	RdRp/Gn/Gc	6.04	7.54	5.10	5.37

**Table 4:** Effect of chemical inhibitors on RVF-VLP delivered minigenome activity.

**Inhibitor**	**Concentration**	**% Activity**
Mock (water)	-	100
Ammonium chloride	5 mM	9.7
	10 mM	1.3
	20 mM	0.5
Guanidine	25 μg/mL	45.8
	50 μg/mL	19.2
	100 μg/mL	22.4
Ribavirin	25 μg/mL	12.8
	50 μg/mL	6.2
	100 μg/mL	4.3
Mock (ethanol)	-	100
Actinomycin D	0.5 μg/mL	13.2
	1.0 μg/mL	6.4
	2.0 μg/mL	5.2
Monensin	0.1 μM	58.2
	1.0 μM	2.1
	10.0 μM	0.2
Mycophenolic acid	1.0 μM	48.6
	10.0 μM	8.9
	100.0 μM	5.8
